# Increased fluid intake and blood pressure in healthy children: a randomized controlled trial. The SPA Project

**DOI:** 10.1007/s00467-025-07054-z

**Published:** 2025-12-22

**Authors:** Gianluigi Ardissino, Laura Viola, Maria Cristina Mancuso, Thomas Ria, Giacomo Tamburini, Daniele Rossetti, Letizia Dato, Andrea Gualtieri, Elena Sacchini, Teresa Nittoli, Chiara Orsenigo, Matteo Vidali, Patrizia Salice

**Affiliations:** 1 Pediatric Nephrology, Dialysis and Transplantation Unit, Centro per la Cura e lo Studio della Sindrome Emolitico-Uremica (Center for HUS Control, Prevention and Management), Fondazione IRCCS Ca’ Granda Ospedale Maggiore Policlinico, Milan, Italy; 2 Pediatric Unit, Ospedale di Stato, ISS, San Marino, San Marino; 3 Division of Pediatrics, Department of Health Sciences, Università del Piemonte Orientale, Novara, Italy; 4 Clinical Pathology Unit, Fondazione IRCCS Ca’ Granda Ospedale Maggiore Policlinico, Milan, Italy

**Keywords:** Blood pressure, Prevention, Hypertension, Water intake

## Abstract

**Background:**

High sodium intake is a key element in the development of hypertension, but strategies aimed at reducing its consumption have had limited impact at a population level. A potential alternative preventive and/or therapeutic opportunity may be represented by increasing kidney sodium excretion.

**Methods:**

The Salus per Aquam Project is a multicenter, prospective, controlled, randomized study investigating whether, in healthy children, increased fluid intake for 1 year can lower blood pressure. Participants were randomized into 2 groups: one was actively encouraged to increase their water intake, especially during school days, while the other group was used as a control. At baseline and after 1 year, blood pressure was determined using multiple office blood pressure measurements. Urinary electrolytes and creatinine were measured in multiple samples at baseline, during the study period, and at the end of the study to provide details on sodium and fluid intake.

**Results:**

One hundred and seventy-five children were enrolled (94 females, 53.7%, median age 8.6 years, IQR:8.4–8.9), but only 145 completed the study. After 12 months, children who were motivated to drink more fluids presented lower median systolic (93 vs. 95 mmHg), diastolic (63 vs. 65 mmHg), and mean (73 vs. 74 mmHg) BP, compared with controls. The median change (ΔMBP, final–baseline) differed significantly between cases and controls, with a between-group median difference of 2 mmHg (Mann–Whitney *p* = 0.018).

**Conclusions:**

An increased fluid intake may prevent the age-related increase of blood pressure in healthy children. We believe this may be due to more efficient sodium excretion by the kidneys. This simple, highly acceptable, inexpensive, and harmless intervention has the potential to prevent or lessen the prevalence of hypertension and associated illnesses both in adults and children.

**Graphical abstract:**

**Figure:**
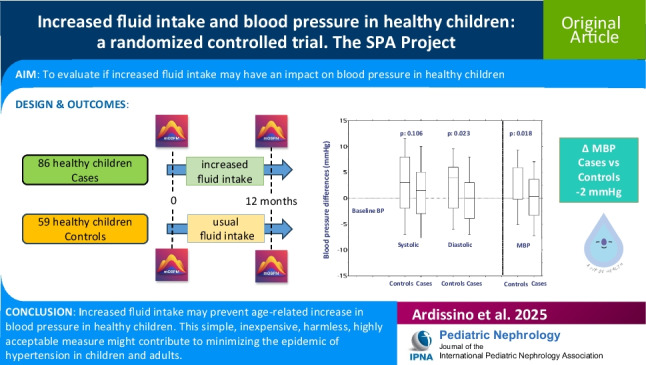
A higher resolution version of the Graphical abstract is available as Supplementary information

**Supplementary Information:**

The online version contains supplementary material available at 10.1007/s00467-025-07054-z.

## Introduction

Hypertension remains a major health problem worldwide, especially in Western countries, given that it is directly responsible for or closely related to several morbidities and a significant proportion of mortality [1, 2].

It is likely that the hypertensive process begins in childhood, considering that children with a family history of hypertension exhibit a significantly higher blood pressure (BP) early in life [3, 4]. The roles of genetic and environmental factors and their interactions are well documented [1–3]. Among the acquired factors, sodium (Na) intake is the one that has been investigated the most, and reduced Na intake remains a cornerstone in the prevention and treatment of all hypertensive conditions [5, 6]. Unfortunately, the strategy of reducing Na intake in the population has been largely unsuccessful, and Na consumption has increased rather than decreased during the last century [7, 8].

Indeed, most of the population is exposed to a very high intake of Na derived from their diet. In adults, the recommended daily Na intake should remain within 2 g/day, whereas in the pediatric population, it should not exceed 1.5 g/day. Despite these indications, almost 80% of children are estimated to far exceed this threshold [9]. Given that kidneys are unable to concentrate urinary Na more than twofold the plasma concentration, it may take additional time to eliminate the Na load derived from the diet in the absence of sufficient water availability to adequately dilute urine [10]. Unfortunately, the increase in Na intake observed in the population is not associated with a proportional increase in fluid consumption [11, 12].

We have previously shown in a retrospective analysis that children who introduce more fluid have a significantly lower BP than those who consume less fluid [13]. Moreover, other studies have highlighted the possible relationship between higher water consumption and BP [14, 15].

Based on these preliminary findings, we investigated whether regular and more generous fluid intake can reduce BP in healthy children with the rationale of increasing the efficiency of kidney Na handling (excretion).

## Methods

The Salus per Aquam (SpA) project is a multicenter, randomized, controlled cohort study aimed at testing the working hypothesis that increased fluid intake may reduce arterial BP in healthy children.

### General procedures

Children attending the 3rd grade of elementary school from 14 classes of 10 different schools in Italy and the San Marino Republic were offered the opportunity to participate in the study as part of school activities. All children whose parents agreed to participate were enrolled, but the analysis was restricted to those born at term, following an uneventful pregnancy, with a normal birth weight. Other reasons for excluding subjects were current or previous kidney disease, chronic systemic disease, acute illnesses, or ongoing treatment at enrolment or at the final assessment. The study obtained the approval of the ethical committee (Comitato Etico Milano Area 2, n. 6098–2022), and it was conducted in accordance with the ethical standards of the Declaration of Helsinki and its amendments.

An informed consent form was administered to parents or legal guardians, and the relevant data (including family and personal medical history of hypertension for both parents and grandparents) were collected by means of a questionnaire. The 10 participating schools were randomly assigned to one of the following groups: (1) increased water intake, and (2) control. The study started in November 2022 and ended in March 2024.

After baseline determinations, the classes and the corresponding pupils who were randomized to the increased water intake group were instructed on the importance of drinking more water than what their thirst requires. The children were also given a water bottle (500 mL) with the study logo to be used during the 12 months ahead to reinforce their motivation to drink. Throughout the study period, teachers of classes randomized to the increased fluid intake group regularly invited children (twice daily) to drink a few sips of water from the provided bottle as a sort of daily habit. Parents were also informed of the usefulness of drinking more water than usual and were asked to provide their children with enough water at school and to have water readily available at home.

### Urine sampling and laboratory determinations

After providing consent, the participants were asked to collect and provide 5 random urine samples taken on 5 different days, which were preserved frozen until they were submitted. Five tubes and written instructions for collecting random urine samples were given to the parents.

The same urine sampling procedure (5 samples on 5 different days within 10 days) was repeated after 6 months from baseline and again at 1 year (just before the end of the study) in all children. Urine samples were collected from treatment participants and control participants on the same days.

Urinary Na (uNa), osmolarity (uOsm), and creatinine (uCr) concentrations were measured in a centralized laboratory (Milan) via standard methods. Urinary parameters at baseline are given as the mean of the 5 urine samples, while those during the study period are the mean of 10 samples (5 taken at 6 months and 5 taken just before the end of the study). To assess relative urine dilution (and by extension, fluid intake), we relied on the mean uCr concentration in the urine samples from each participant. In addition, the uNa-to-uCr ratio (uNa/uCr) was calculated as the mean of multiple samples (5 at baseline and 10 during the study) to explore Na intake in the two groups of subjects.

### Anthropometry and BP measurement

After baseline urine sampling and during school hours, all the participants underwent anthropometric measurements (height and weight) and multiple office BP measurements (mOBPMs). Ten BP readings were taken on the nondominant arm at 3 minutes intervals using an automated oscillometric device (Omron M3, Omron Healthcare, Hoofddorp, The Netherlands). The mOBPM methodology is described in greater detail elsewhere [16].

MOBPMs showing more than a 15% coefficient of variation were deemed unreliable and discarded. The mean systolic and diastolic BP were measured and normalized for age, sex, and height according to the 2017 Guideline for Screening and Management of High Blood Pressure in Children and Adolescents [17]. The normalization procedure included the conversion of BP expressed in mmHg into z-score based on data derived from the above-mentioned reference tables.

Anthropometry and mOBPM were repeated 1 year after baseline. All anthropometric measurements and mOBPMs (baseline and final) were performed on the same day in both treatment participants and control participants.

### Statistical analysis

Statistical analyses were carried out using SPSS software v.27 (SPSS Inc., Chicago, IL, USA) and R Language v.4.3.2 (R Foundation for Statistical Computing, Vienna, Austria). The normality of the distribution was assessed by a q-q plot and the Shapiro–Wilk test. Quantitative variables are presented as medians and interquartile ranges (IQRs), whereas qualitative variables are presented as absolute or relative frequencies. Associations between qualitative variables were performed by Fisher’s exact test.

Differences between independent and paired groups were assessed with the nonparametric Mann‒Whitney test and the Wilcoxon signed rank test, respectively. The differences between treatment participants and control participants for variables measured at the end of the study were evaluated by the analysis of covariance (ANCOVA) after adjusting for the levels of the same variables (covariates) measured at the beginning of the study.

## Results

One hundred and seventy-five healthy children (94 females, 53.7%) with a median age of 8.6 years (IQR: 8.4–8.9) were enrolled, but only 145 contributed to the analysis by successfully completing the study protocol. The reasons for being excluded from the analysis were the following (in brackets the distribution according to study group control/treatment): 3 (2/1) chronic diseases, 7 (2/5) acute illnesses at baseline or at the final evaluation, 8 (3/5) urine samples not provided, 12 (7/5) coefficient of variation (CV) > 15% in the systolic or diastolic mOBPM at baseline or final determination.

Fifty-nine control participants (52.5% females) and 86 treatment participants (61.6% females) were available for the analysis. The 2 groups were comparable in terms of demography, systolic, diastolic, and mean BP values at baseline (Table 1) except for a significantly higher percentage of the treatment group having a positive family history of hypertension (10.3% vs. 3.8%; *p* = 0.04).

**Table 1:** Baseline characteristics, blood pressure, and laboratory parameters in children addressed to increased fluid intake and controls

	**Cases (*n*:86)**	**Controls (*n*:59)**	***p***
Sex, M/F	33/53	28/31	0.307
Age, years	8.6 (8.4–8.9)	8.7 (8.3–8.9)	0.982
Weight, kg	30 (26–35)	30 (26–35)	0.866
Weight SDS	0.59 (−0.19 to 1.41)	0.65 (−0.07 to 2.03)	0.566
Height, cm	133 (128–137)	132 (128–138)	0.847
Height SDS	0.73 (−0.07 to 1.36)	0.50 (0.07 to 1.17)	0.775
BMI, kg/m^2^	16.7 (15.1–18.9)	17.0 (15.4–19.3)	0.720
Family history of HPT			
Parents, *n*. (%)*	16/155 (10.3)	4/104 (3.8)	0.040
Grandparents, *n*. (%)*	61/166 (36.7)	11/38 (28.9)	0.364
Systolic BP, mmHg	93 (87–98)	93 (86–97)	0.893
Systolic BP SDS	−0.28 (−0.72 to −0.06)	−0.25 (−0.60 to −0.08)	0.886
Diastolic BP, mmHg	62 (59–67)	61 (58–66)	0.412
Diastolic BP SDS	0.22 (−0.19 to 0.56)	0.09 (−0.15 to 0.40)	0.433
Mean BP, mmHg	73 (69–77)	73 (64–76)	0.539
Mean BP SDS	−0.12 (−0.22 to 0.29)	−0.07 (−0.29 to 0.25)	0.577
uCr**, mg/dL	104 (78–121)	101 (86–122)	0.546
uNa/uCr**, mEq/mg	1.45 (1.20–1.71)	1.48 (1.31–1.69)	0.533
uOsm, mOsm/kg	103.5 (80.5–130.3)	115.5 (92.3–139.3)	0.034^#^

Legend. *HPT* hypertension, *BP* blood pressure, *SDS* standard deviation score, *uCr* urinary creatinine, *uNa* urinary sodium, *uOsm* urinary osmolarity

*Number of hypertensive parents or grandparents over available data

**Mean of 5 baseline samples

Data are expressed as frequencies (%) or as medians and interquartile range (1° and 3° quartile)

^#^Cases and controls were not significantly different for final uOsm after taking into account basal uOsm by ANCOVA (*p* = 0.450)

**Fig. 1:**
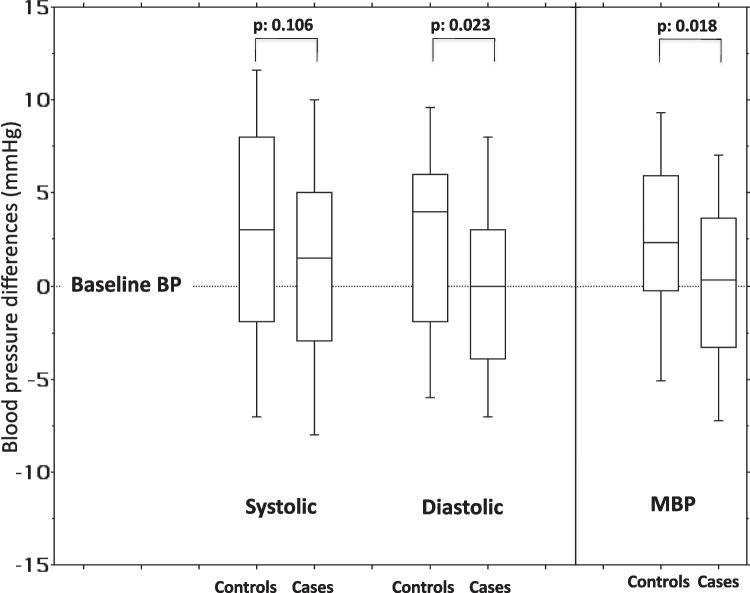
Changes in blood pressure compared to baseline values in children exposed to increased water intake (*n* = 86) vs. controls (*n* = 59), 1 year apart. BP, blood pressure; MBP, mean arterial blood pressure

In control participants, as expected for the age increase, from baseline to the final measurement, raw systolic and diastolic BP values significantly increased from 93 to 95 mmHg (*p* = 0.002) and from 61 to 65 mmHg (*p* = 0.010), respectively. Among treatment participants, both the systolic and diastolic baseline values were not different from those measured at the end of the study: 93 vs. 93 mmHg (*p* = 0.101) and 62 vs. 63 mmHg (*p* = 0.957). Accordingly, in the control group, MBP values differed significantly between baseline and final measurements (73 vs. 74 mmHg, *p* = 0.001), whereas no significant change was observed among cases (73 vs. 73 mmHg, *p* = 0.619) (Tables 1 and 2). The change in BP values from baseline to the end of the study was further evaluated and calculated separately in cases and controls as (final-basal) delta. Compared with treatment participants, control participants showed a significantly greater diastolic BP delta (final-baseline) than treatment participants, both for raw values: + 4 vs. 0 mmHg (*p* = 0.023) (Fig. 1) and standardized values: 0.20 vs. −0.09 (*p* = 0.018). Conversely, no significant difference was observed for delta values (final-baseline) of systolic BP: 3 vs. 1.5 mmHg (*p* = 0.106) and −0.01 vs. 0.03 (*p* = 0.278), for raw and standardized values, respectively. The same was true for mean BP (Tables 1 and 2). Final-basal deltas for MBP, among controls and cases, were 2.3 vs 0.3 mmHg (*p* = 0.018) and 0.01 vs −0.07 (*p* = 0.164), for raw and standardized values, respectively.

**Table 2:** Anthropometry, blood pressure, and laboratory findings in children addressed to increased fluid intake and controls at final evaluation

	**Cases (*n*:86)**	**Controls (*n*:59)**	***p***
Weight, kg	35 (28–39)	33 (29–39)	0.987
Weight SDS	0.65 (−0.14 to 1.28)	0.43 (−0.10 to 1.55)	0.829
Height, cm	138 (133–143)	138 (133–142)	0.622
Height SDS	0.67 (−0.08 to 1.28)	0.45 (−0.04 to 1.01)	0.612
BMI, kg/m^2^	17.3 (15.8–20.0)	17.2 (15.8–19.9)	0.918
Systolic BP, mmHg	93 (89–99)	95 (90–100)	0.251
Systolic BP SDS	−0.13 (−0.67 to −0.06)	−0.50 (−0.87 to −0.05)	0.172
Diastolic BP, mmHg	63 (58–67)	65 (59–70)	0.129
Diastolic BP SDS	0.08 (−0.25 to 0.57)	0.35 (−0.10 to 0.80)	0.059
Mean BP, mmHg	73 (68–76)	74 (69–80)	0.115
Mean BP SDS	−0.04 (−0.35 to 0.25)	0.03 (−0.27 to 0.27)	0.326
uCr*, mg/dL	97 (77–120)	110 (79–128)	0.621
uNa/uCr*, mEq/mg	1.43 (1.12–1.82)	1.49 (1.23–1.79)	0.439
uOsm, mOsm/kg	97.0 (79.0–114.0)	104.5 (79.8–122.3)	0.157

Legend. *BP* blood pressure, *SDS* standard deviation score, *uCr* urinary creatinine, *uNa* urinary sodium, *uOsm* urinary osmolarity

*Mean of 10 samples during the study period

Data are expressed as frequencies (%) or as medians and interquartile range (1° and 3° quartile)

To confirm these trends, the analysis of covariance (ANCOVA) was used to evaluate the difference in final BP values between treatment participants and control participants after adjustment for baseline values (Table 3). A statistically significant difference was observed in final diastolic BP between treatment participants and control participants, both considering raw (adjusted mean 62.6 vs. 64.7 mmHg; *p* = 0.022) and standardized values (adjusted mean 0.136 vs. 0.334; *p* = 0.013). Systolic BP values in treatment participants and control participants for both raw (adjusted mean 93.7 vs. 95.4 mmHg; *p* = 0.090) and standardized values (adjusted mean −0.477 vs. −0.350; *p* = 0.140), although different, did not reach statistical significance. Final MBP values between treatment and control participants were significantly different when considering raw values (adjusted mean 73.0 vs. 74.9 mmHg; *p* = 0.018), whereas no significant difference was found for standardized values (adjusted mean −0.028 vs. 0.064; *p* = 0.159) (Table 3).

**Table 3:** ANCOVA analysis to assess the difference in final blood pressure between cases and controls after adjustment for baseline values

Variables	ANCOVA analysis
Final systolic BP	Adjusted for basal systolic BP
mmHg	*p* = 0.090
SDS	*p* = 0.140
Final diastolic BP	Adjusted for basal diastolic BP
mmHg	*p* = 0.022*
SDS	*p* = 0.013*
Final mean BP	Adjusted for basal mean BP
mmHg	*p* = 0.018*
SDS	*p* = 0.159

Legend. *BP* blood pressure, *SDS* standard deviation score; *statistically significant

There was no significant difference between groups for the results of urine determinations at 6 months and 1 year; thus, these results were pooled together (Table 2). Urinary Cr values decreased in treatment participants and increased in control participants, whereas uNa/uCr ratios remained unchanged in both groups.

Moreover, despite the increased water intake reported by teachers in treatment participants, no significant difference in urinary osmolarity was detected after the intervention both as absolute value or as percent difference within and between groups (Tables 1 and 2). A significant case–control difference in final uOsm emerged in the univariate comparison (*p* = 0.034, Table 2); however, after adjustment for baseline uOsm, using ANCOVA, the difference was not confirmed (*p* = 0.450).

## Discussion

Following a previous cross-sectional observational study suggesting an inverse correlation between urine dilution and BP in healthy children [13], in the present prospective interventional study, we tried to test the working hypothesis that an increased fluid intake can lower BP. The results seem to confirm this hypothesis: a few additional sips of water, introduced regularly for 1 year, seem to have blunted the age-related increase in BP. In greater detail, children exposed to a daily extra intake of water for 1 year on the order of 120 ml (equivalent to 6% of daily fluid allowance for weight) showed a mean BP 2 mmHg lower than that of the control participants. The amount of extra water was estimated from the change in uCr based on our previous investigation on the relationship between uCr and urine dilution [18].

Our interpretation of these results, although not indisputable, is that the increased availability of water may enhance the ability of the kidney to excrete the excessive Na intake that typically characterizes the current diet among children. This is consistent with the well-established mechanism of “pressure natriuresis” postulated by Guyton et al. more than 50 years ago [19].

To precisely measure uNa excretion by means of 24 h urine collections (the standard of care) to demonstrate this explanation was clearly unfeasible in the present study setting (175 pupils enrolled in schools). Moreover, few urine collections (at baseline and during the study period) would have likely been insufficient to document a change in Na excretion (as well as in urine volume) given the high variability in Na (and fluid) intake day by day and the multiple sources of error typically affecting urine collection in children (measurement of volume, of time, lost micturition, enuresis, etc.). Thus, the interpretation of our results must rely mainly upon physiological notions. Moreover, the metabolism of Na and water and their influence on BP are complex. Stored Na (in bones, muscles, skin, and endothelia surface layers, bound to proteins or to glycosaminoglycans), although osmotically inactive, is in constant dynamic equilibrium with serum Na and, consequently, with urinary Na. However, stored Na, which forms a dynamic third compartment, is not influenced by sudden increases or decreases in Na intake and/or excretion, but rather by persistent and long-lasting changes [11, 12, 20]. For this reason, traditional techniques used to quantify Na intake (including uNa measured on 24 h urine collection) may not accurately reflect long-lasting changes in Na metabolism, but can only reflect changes in the hours immediately following events that disrupt the steady state [10]. Therefore, although 24 h urine collection remains the most accurate way to evaluate Na balance, we decided to use the uNa/uCr ratio (as the mean of multiple samples, 5 at baseline and 10 during the study, all collected within a very short period of time) only in order to support the methodological assumption that cases and controls did not significantly differ as to their dietary habits (with regard to Na intake) nor significantly changed them during the study period, also based on previous work done by our group in support of this surrogate approach [21].

Another possible explanation of our findings may rely on the suppression of vasopressin production by the extra intake of fluids, given that this hormone is among the most potent vasopressors [22]. A high intake of salty food, however, stimulates vasopressin, encouraging water retention even as it hampers the body’s ability to excrete sodium, leading to fluid retention. This trade-off favoring water conservation at the expense of sodium excretion has been proposed as an outcome of natural selection. The reasoning is that acute dehydration presents immediate and severe health threats, whereas the detrimental effects of Na retention (such as hypertension) typically appear only over the long term, well after the reproductive years [23].

However, even in the case of a potential role of vasopressin, the mechanism can only be hypothesized but cannot be proven in our experimental setting, and other studies will be necessary to identify the specific role played by the suppression of this hormone in the observed effect.

**Fig. 2:**
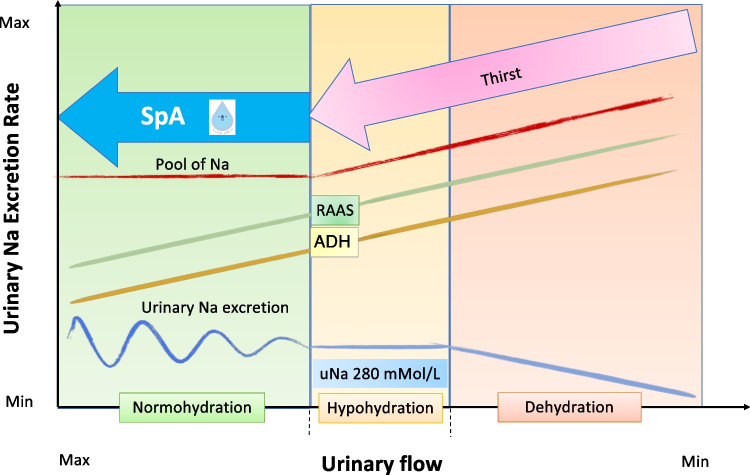
Simplified schematic representation of the relationship between dietary sodium intake, hydration status, fluid intake, blood pressure control and the mechanism underlying the effect of the intervention in the SpA project. In the area of normo-hydration, urinary sodium excretion (blue line) swings according to sodium intake. In the area of hypo-hydration it is likely that the threshold of the maximal urinary sodium concentration of 280 mEq/L is reached because of the reduced availability of water and beyond this concentration the kidney cannot increase sodium excretion further. In the area of dehydration, urinary sodium excretion decreases; thus sodium tends to accumulate with a consequent increase in the pool of body sodium (red line) and the activation of neuroendocrine compensatory mechanisms, all involving blood pressure homeostasis. In the area of hypohydration, and even more so, in that of dehydration, thirst will eventually restore the equilibrium of the system. An increased intake of water, regardless of thirst (as obtained in the SpA project) keeps the system leftward with a consequent more efficient sodium handling by the kidney. SpA, Salus per Aquam project; Na, sodium; uNa, urinary sodium; RAAS, renin-angiotensin-aldosterone system; ADH, anti-diuretic hormone

A schematic representation of the relationship between sodium intake, hydration, fluid intake, BP control, and the hypothesized mechanism underlying the intervention within the SpA project is provided in Fig. 2.

The hydration status of normal subjects is the balance between intake (including fluids contained in foods) and outputs (via urine, skin, breath, etc.), and cannot be explored without taking into consideration all these and other (temperature, physical exercise, etc.) variables. Urine volume and urine dilution are the result of all these interplaying factors: a well-hydrated subject will excrete the extra fluid in urine, leading to larger urine volume and more diluted urine, while if dehydrated, the urine volume will be reduced with higher concentration. This is the reason why, in the present study, it was decided to investigate fluid intake by urine dilution rather than by fluid intake.

However, the use of uCr as a surrogate marker of urine dilution in healthy prepubertal children of similar ages (with similar muscle masses) deserves further comment. Better hydration is associated with an increase in urine volume and urine dilution, thus reducing the uCr concentration. The inverse correlation between urine dilution and uCr is well documented, and, in fact, uCr is frequently used in pediatric clinical practice to adjust most urine parameters for varying urine concentrations [18, 24, 25].

In our analysis, the median uCr concentration during the study period decreased in treatment participants (−6.7%) compared with control participants (+ 8.9%). Although the difference did not reach statistical significance, the group of children with increased water intake (thus with more diluted urine) did not exhibit the physiological increase in uCr expected with growth, whereas this increase was observed in the control participants (for the growth-related increase in muscle mass and Cr generation) [26, 27].

With respect to BP, in 1 year and under normal conditions, a growing child shows an increase in mean BP on the order of 2 mmHg [15]. This increase was observed only in the control group, whereas in the group exposed to increased water intake, no increase in BP was detected. The noted difference in BP might seem insignificant when viewed as raw figures. However, it must also be considered that modest BP differences in childhood are amplified in adulthood, as demonstrated by BP tracking [28, 29]. In addition, according to data from the Framingham Heart Study and NHANES II, even a modest 2 mmHg decrease in population blood pressure has the potential to reduce hypertension by 17% and coronary heart disease by 6% [30].

Interestingly, our findings seem to confirm the effect of increased fluid intake on diastolic rather than on systolic BP, as shown in the previous observational study [13]. It may be worth noting that the approach used to measure BP in the present study (mOBPM) is not formally validated; however, it only consists of multiple measurements that are likely to provide a more reliable BP evaluation compared with that obtained by fewer measurements. In addition, multiple determinations significantly reduce the number of subjects needed to reach the desired statistical power by reducing the high variability of BP readings in children. Finally, although BP values were standardized to tables created using auscultatory BP values, given the comparative nature of the analysis and the homogeneous age of subjects, we believe that results are not affected by the standardization approach.

A possible further limitation of our study is that the groups were not well balanced as to sex distribution, with males constituting 38% of the treatment group compared to 47% of the control group. This imbalance may have affected BP at study completion as differences in BP trajectories between the sexes may begin prior to the onset of puberty [31].

Moreover, a significantly higher percentage of the treatment group had a positive family history for hypertension as compared with the control group (Table 1). It is possible that a lower Na intake might be provided in these families because they might find renewed interest in adhering to a previously recommended reduced salt diet. However, this remains a theoretical bias, and based on the available data (with their limitations), there was no evidence of a lower Na intake in the treatment group. In addition, a positive family history of hypertension involving the treatment group is expected to lead to increased BP compared with controls, not to a decrease as observed.

Another limitation of the present work is that the multiple urine samples did not capture a statistically significant urine dilution (in terms of uCr concentration and/or urinary osmolarity; Tables 1 and 2) in the group subjected to an intervention to increase fluid intake. UOsm (considered more reliable for investigating urine dilution) decreased in both groups, and our conclusions must solely rely upon the methodological aspect that during lessons, teachers did invite pupils in the intervention group to drink those few sips of water every day as per the protocol. The discrepancy between changes in uCr and in uOsm might be explained by the hypothesis that 10 spot urine samples, over a study period of 1 year, were inadequate to explore changes in urine dilution.

## Conclusions

The Western diet provides an excess of Na intake that is not compensated for by a proportionate increase in fluids. Healthy lifestyle habits should include adequate hydration. This simple, inexpensive, highly acceptable, and harmless measure may help in counteracting excess Na intake and its detrimental effect on BP. The implementation of this preventive strategy, as early as the pediatric age, might be an additional tool to address the hypertension epidemic in teenagers, adults, and the elderly. Particularly in school settings, it is recommended that children have free access to drinks and that the common practice of forbidding them to drink during lessons be abandoned. Further studies on this issue are needed (perhaps with the prescription of a precise amount of extra fluids) to confirm our findings, explain the mechanisms underlying them, and prove the long-term sustainability of increased water intake.

## Supplementary Information

Below is the link to the electronic supplementary material.

Graphical abstract (PPTX 6.42 MB)

## Data Availability

Data are available upon reasonable request.

## Abbreviations

BP: Blood pressure
Na: Sodium
SpA: Salus per Aquam
uNa: Urinary sodium
uK: Urinary potassium
uCr: Urinary creatinine
mOBPM: Multiple Office Blood Pressure Measurement
IQR: Interquartile range
