# Supplementary material for: Increased fluid intake and blood pressure in healthy children: a randomized controlled trial. The SPA Project
Source: Pediatr Nephrol. 2025 Dec 22;41(5):1433–41. doi: 10.1007/s00467-025-07054-z (PMC13009042; doi:10.1007/s00467-025-07054-z)
Supplement: Supplementary file 1 — Graphical abstract (PPTX 6.42 MB) [file 467_2025_7054_MOESM1_ESM.pptx]

## Slide 1
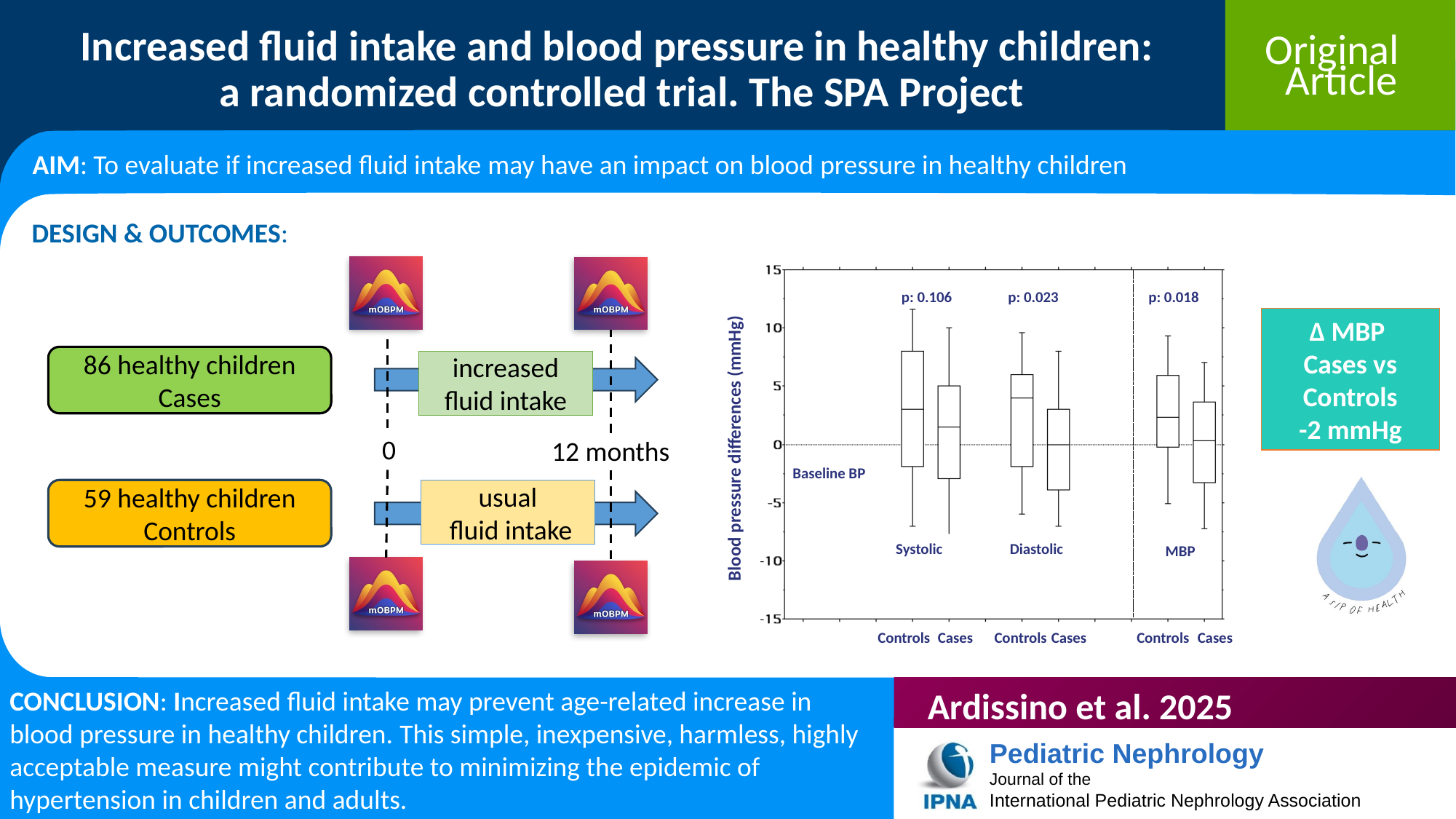

Increased fluid intake and blood pressure in healthy children:
a randomized controlled trial. The SPA Project
AIM: To evaluate if increased fluid intake may have an impact on blood pressure in healthy children
DESIGN & OUTCOMES:
p: 0.106
p: 0.023
p: 0.018
Δ MBP
Cases vs Controls
-2 mmHg
86 healthy children
Cases
increased fluid intake
Blood pressure differences (mmHg)
0
12 months
Baseline BP
usual
 fluid intake
59 healthy children
Controls
Systolic
Diastolic
MBP
Controls
Cases
Controls
Cases
Cases
Controls
Ardissino et al. 2025
CONCLUSION: Increased fluid intake may prevent age-related increase in blood pressure in healthy children. This simple, inexpensive, harmless, highly acceptable measure might contribute to minimizing the epidemic of hypertension in children and adults.
